# Longitudinal Inpatient Trajectories After Splenic Artery Embolization in Cirrhosis: Real-World Evidence from Kazakhstan

**DOI:** 10.3390/jcm15114337

**Published:** 2026-06-03

**Authors:** Ainur Doszhan, Niyaz Malayev, Abai Baigenzhin, Nina Tostanovskaya, Anuar Abdikarimov, Kristina Pavlova, Assyltay Nauryzbayeva, Balzhan Abzhaparova, Gulsara Imambaeva

**Affiliations:** 1 JSC National Scientific Medical Center, 010009 Astana, Kazakhstan; ainurdoszhan8@gmail.com (A.D.); niyaz.malayev@gmail.com (N.M.); abai.baigenzhin@outlook.com (A.B.); nina.tostanovskaya@gmail.com (N.T.); a.abdikarimov@nnmc.kz (A.A.); kristina1pavlova@gmail.com (K.P.); lecarassyltay@gmail.com (A.N.); 2 National Scientific Center of Surgery Named After A.N. Syzganov, 050004 Almaty, Kazakhstan; 3 Department of Internal Medicine No. 2, Astana Medical University, 010000 Astana, Kazakhstan; imambayeva7g@gmail.com

**Keywords:** cirrhosis, splenic artery embolization, portal hypertension, hypersplenism, hospitalization, real-world study, liver disease, clinical outcomes

## Abstract

**Background:** Splenic artery embolization (SAE) is commonly used in cirrhosis to manage hypersplenism and cytopenia. However, its longer-term clinical impact beyond hematologic parameters remains insufficiently characterized. **Aim:** To characterize longitudinal inpatient trajectories, clinical patterns, and follow-up features after SAE in patients with cirrhosis treated at a tertiary referral center in Kazakhstan. **Methods:** This retrospective single-center study included 149 patients with cirrhosis who underwent SAE. Clinical, laboratory, and imaging data were collected across sequential inpatient episodes. Outcomes included longitudinal patterns of hospitalization, laboratory trends, and baseline factors associated with a favorable clinical course. Subsequent hospitalization was defined as any inpatient episode following the index SAE admission, regardless of whether it was planned or unplanned. **Results:** During follow-up, 59.1% of patients had a second inpatient episode, with progressively fewer patients contributing to later observations. Liver disease severity remained largely stable, with no significant changes in Child–Pugh distribution. Portal hypertension manifestations, including varices and splenomegaly, remained highly prevalent, while recurrent variceal bleeding was relatively uncommon. Laboratory parameters demonstrated modest changes without sustained statistically significant improvement, and the number of recorded inpatient episodes decreased across sequential follow-up. Relatively stable documented follow-up trajectories were more frequently observed in patients with preserved liver function (Child–Pugh A), absence of ascites, and higher albumin levels. The most common causes of subsequent hospitalization were ascites and hepatic decompensation (38.9%) and varices without bleeding (26.2%), while documented major procedure-related complications were infrequent. **Conclusions:** In this retrospective single-center cohort, predominantly coil-based proximal or selective SAE demonstrated an acceptable documented major complication profile in selected patients with cirrhosis and portal hypertension. Because no untreated control group was available, the findings should be interpreted as descriptive real-world longitudinal data rather than causal evidence of reduced hospitalization burden.

## 1. Introduction

Liver cirrhosis remains a major global health challenge and is one of the leading causes of liver-related morbidity, premature mortality, and healthcare utilization worldwide [1]. Recent Global Burden of Disease analyses demonstrate that the worldwide burden of chronic liver disease continues to rise, driven by both increasing prevalence and persistent mortality [2]. In 2017, an estimated 5.2 million individuals were living with cirrhosis or advanced chronic liver disease globally, while cirrhosis accounted for approximately 1.48 million deaths in 2019. In parallel, cirrhosis contributes substantially to disability-adjusted life-years (DALYs), reflecting its combined impact on early death and prolonged disability [3]. Importantly, the epidemiologic profile of cirrhosis is changing: although the burden attributable to chronic Hepatitis B and Hepatitis C has declined in several regions because of vaccination and antiviral therapy [4,5], cirrhosis related to alcohol-associated liver disease and metabolic dysfunction-associated steatotic liver disease continues to increase rapidly [6,7].

Once clinically significant portal hypertension develops, the clinical course of cirrhosis changes substantially [8]. Portal hypertension underlies many of the most serious complications of advanced liver disease, including gastroesophageal varices, ascites, spontaneous bacterial peritonitis, renal dysfunction, hepatic encephalopathy, and recurrent hospitalization [9,10]. The transition from compensated to decompensated cirrhosis is associated with a marked decline in survival and a major increase in healthcare utilization [11]. This issue is particularly relevant for Central Asia, where recent global burden analyses identified the region as having one of the highest mortality burdens from alcohol-related cirrhosis, with an age-standardized mortality rate of 11.63 per 100,000 population in 2021 [12]. In Kazakhstan, national epidemiological data have shown a steady increase in the prevalence of liver fibrosis and cirrhosis between 2015 and 2023, with further growth projected through 2030 [13,14]. At the same time, only 474 liver transplantations were performed nationally between 2012 and 2023, while 364 patients remained on the waiting list without transplantation, underscoring the gap between disease burden and access to definitive treatment [13].

Among the complications of portal hypertension, hypersplenism remains clinically important yet frequently underrecognized [15,16]. Congestive splenomegaly may lead to thrombocytopenia, leukopenia, and anemia through splenic sequestration and altered hematopoiesis [17]. Of these abnormalities, thrombocytopenia has particular practical significance because low platelet counts may delay endoscopic treatment, surgery, locoregional oncologic procedures, liver biopsy, and antiviral therapy while increasing dependence on transfusion support or thrombopoietin receptor agonists [18]. As a result, hypersplenism often becomes a barrier to optimal multidisciplinary management rather than merely a laboratory abnormality [19].

Therapeutic options for clinically significant hypersplenism in cirrhosis remain limited [20]. Although splenectomy can effectively correct hypersplenism, its use in cirrhosis is limited by perioperative risk related to portal hypertension, hepatic dysfunction, and postoperative portal venous thrombosis [21]. Consequently, minimally invasive alternatives have gained increasing interest. Splenic artery embolization, including partial splenic embolization techniques, reduces splenic arterial inflow and functional sequestration while preserving residual splenic tissue [22]. Contemporary studies indicate that SAE can improve platelet counts in many patients with cirrhosis-associated hypersplenism, although the magnitude and durability of response vary substantially according to embolization extent, baseline liver function, and follow-up duration. Recent cohorts and observational studies have consistently reported significant post-procedural hematologic improvement after partial splenic embolization [23,24]. Most adverse events are mild and self-limited, particularly post-embolization pain and fever, whereas serious complications such as splenic abscess or portal vein thrombosis are less frequent [25].

Importantly, most available studies have focused on short-term hematologic response or procedural safety [26,27]. Considerably less is known about longer-term real-world outcomes after SAE, including whether the procedure is associated with differences in longitudinal hospitalization patterns. This evidence gap is particularly relevant in Kazakhstan and the wider Central Asian region, where cirrhosis prevalence is rising but published outcome data on interventional management remain scarce [13].

Accordingly, the present study aimed to characterize longitudinal inpatient trajectories, clinical patterns, and follow-up features after SAE in patients with cirrhosis treated at a tertiary referral center in Kazakhstan, using an episode-based inpatient follow-up framework. Specifically, we assessed the pattern and burden of subsequent inpatient episodes, the timing and causes of later hospitalizations, the evolution of clinical severity markers, laboratory trends, and baseline factors associated with a favorable clinical course.

## 2. Materials and Methods

### 2.1. Study Design and Setting

This retrospective observational cohort study was conducted at the JSC National Scientific Medical Center in Astana, Kazakhstan, a tertiary referral center providing specialized hepatology, gastroenterology, surgical, and interventional radiology care. The study evaluated clinical outcomes of patients with liver cirrhosis who underwent splenic artery embolization (SAE) between January 2021 and December 2025.

The hospitalization during which SAE was performed was considered the index admission. Subsequent inpatient encounters documented in the institutional database were used for longitudinal follow-up and assessment of post-procedural outcomes.

### 2.2. Ethical Approval

The study protocol was reviewed and approved by the Local Ethics Committee of JSC National Scientific Medical Center (protocol 108/KI-102 on 2 December 2025) prior to data analysis. The approval covered the complete retrospective review of clinical records and institutional database extraction for the study period from January 2021 through December 2025. The investigation was performed in accordance with the principles of the Declaration of Helsinki and applicable national regulatory standards.

Due to the retrospective design and use of anonymized data, the requirement for informed consent was waived by the local ethics committee.

### 2.3. Study Population

The study included consecutive adult patients with established liver cirrhosis who underwent SAE during the study period. Cirrhosis had been diagnosed before intervention on the basis of integrated clinical, laboratory, imaging, and endoscopic findings consistent with routine hepatology practice.

Patients were considered for SAE in the presence of clinically significant hypersplenism, thrombocytopenia affecting treatment strategy, symptomatic splenomegaly, recurrent portal hypertension-related complications, or when a minimally invasive alternative to surgery was clinically preferable.

### 2.4. Eligibility Criteria

Eligibility criteria, including inclusion and exclusion parameters, are summarized in Table 1.

### 2.5. Splenic Artery Embolization Procedure

All patients underwent SAE during the index hospitalization. Procedures were performed by experienced interventional radiologists in a dedicated angiography suite under sterile conditions and fluoroscopic guidance.

After vascular access had been obtained, angiographic assessment of splenic arterial anatomy was performed, followed by selective catheterization of the splenic artery or its branches according to individual anatomy and procedural objectives.

Embolization was predominantly coil-based and was performed using detachable or pushable coils. The embolization level was determined by splenic arterial anatomy, the intended reduction in splenic arterial inflow, baseline hepatic reserve, platelet count, bleeding risk, and operator judgment. Because embolization was primarily coil-based, the procedure most often corresponded to proximal or selective splenic artery embolization rather than particle-based parenchymal partial splenic embolization.

The extent of embolization was individualized in order to reduce splenic perfusion and hypersplenism while limiting the risk of extensive infarction or severe post-embolization complications. In selected patients, repeat or staged SAE was performed during a subsequent hospitalization when clinically indicated because of persistent or recurrent hypersplenism, insufficient hematologic response, residual splenic perfusion, or ongoing portal hypertension-related morbidity.

Target infarction volume was not routinely predefined. The achieved infarcted splenic volume was not systematically quantified because follow-up contrast-enhanced CT was not uniformly performed in all patients, and detailed angiographic quantification of segmental infarction was not consistently available in retrospective procedural records.

Routine post-procedural CT was not performed in every patient because of the retrospective real-world design, regional follow-up logistics, radiation exposure, contrast-related considerations, and unequal access to CT imaging across referring regions. Post-procedural imaging was performed selectively when clinically indicated, particularly in patients with persistent fever, severe pain, suspected splenic abscess, suspected portal or splenic vein thrombosis, pancreatitis, or other signs of complications. Following intervention, patients received standard post-procedural monitoring and supportive inpatient care according to institutional protocols. Analgesic and antibiotic therapy were administered according to routine institutional post-procedural protocols and individual clinical indications.

### 2.6. Longitudinal Data Structure and Follow-Up

The unit of longitudinal observation was a hospitalization episode. Sequential episodes after the index SAE admission included: second recorded hospitalization (which could represent repeat SAE, inpatient follow-up, or readmission); later hospitalizations related to cirrhosis progression, portal hypertension complications, repeat intervention, or other clinical indications.

This episode-based framework was used to characterize real-world inpatient trajectories after SAE. Importantly, some second or later hospitalization episodes represented planned or staged repeat SAE procedures rather than unplanned readmissions. Therefore, all subsequent inpatient encounters were analyzed within a unified episode-based framework without assuming that they reflected adverse clinical events.

Follow-up was influenced by the referral structure of care in Kazakhstan. Many patients were referred from different regions and continued subsequent observation at local healthcare facilities after discharge from the tertiary center. As a result, the timing and completeness of follow-up differed substantially between patients. The study therefore focused on documented inpatient episodes within the institutional database and available follow-up records rather than assuming uniform scheduled follow-up for the entire cohort.

### 2.7. Data Collection and Management

Clinical data were retrospectively extracted from the electronic medical record system, hospitalization registry, procedural reports, discharge summaries, and follow-up documentation. Final retrospective data extraction and database verification were completed in December 2025. The study database was assembled, coded, cleaned, and verified using Microsoft Excel. Collected variables included: demographic characteristics; hospitalization dates; liver disease severity indicators (Child–Pugh class and, where available, MELD-Na); manifestations of portal hypertension; imaging markers of splenic and portal venous changes; laboratory parameters (platelet count, leukocyte count, erythrocyte count, and albumin); repeat SAE procedures; causes of subsequent hospitalization; and procedure-related complications.

### 2.8. Clinical Definitions and Outcomes

Severity of liver disease was assessed using routinely documented Child–Pugh categories (A, B, or C). Where available, MELD or MELD-Na values were reviewed.

Subsequent hospitalization was defined as any inpatient episode occurring after discharge from the index SAE admission. Importantly, some subsequent inpatient episodes represented planned or staged repeat SAE procedures rather than unplanned readmissions. Hospitalizations were clinically categorized as related to: ascites or hepatic decompensation; variceal disease; acute variceal bleeding; hypersplenism-related indications; repeat SAE; and other causes.

The primary endpoint was descriptive characterization of longitudinal inpatient trajectories after index SAE, assessed according to the number, sequence, timing, and clinical causes of subsequent hospitalization episodes during follow-up.

Secondary endpoints included hematologic trajectories after SAE, need for repeat or staged SAE, time to first subsequent hospitalization, causes of later admissions, documented variceal bleeding, longitudinal changes in clinical severity markers, and documented major procedure-related complications.

Hematologic response was assessed descriptively using within-patient platelet and leukocyte dynamics compared with baseline values before the index procedure. Platelet and leukocyte trends were evaluated across available post-procedural inpatient episodes and follow-up visits documented in the study database.

Because follow-up intervals and the number of recorded visits differed substantially between patients, hematologic response was analyzed using available paired measurements at each documented episode rather than fixed uniform time points.

Ascites progression and Child–Pugh class deterioration were not included in the formal definition of treatment response because these parameters may reflect the natural course of cirrhosis rather than the direct effect of SAE. Child–Pugh class, ascites, albumin level, and variceal status were therefore analyzed descriptively as baseline and follow-up clinical characteristics rather than as components of a composite response endpoint. Because this was a retrospective single-arm before–after study, all outcome analyses were interpreted descriptively. Patients were not assumed to have a favorable response solely because of missing follow-up data; exploratory trajectory assignment was based only on documented laboratory values and clinical events available in the medical records. Patients with insufficient longitudinal follow-up information were not assigned to exploratory trajectory subgroup comparisons.

### 2.9. Statistical Analysis

All analyses were performed using IBM SPSS Statistics (version 26.0). Normally distributed continuous variables were summarized as mean ± standard deviation (SD), whereas non-normally distributed data were reported as median with interquartile range (IQR). Categorical variables were expressed as counts and percentages. Between-group comparisons were performed using Student’s *t*-test or Mann–Whitney U test, as appropriate. Categorical variables were compared using Pearson’s chi-square or Fisher’s exact test. Sequential hospitalization patterns were analyzed descriptively across recorded episodes. Time to first subsequent hospitalization was defined as the interval between discharge from the index SAE admission and the next documented inpatient episode among patients with complete paired dates.

All tests were two-sided, and *p* < 0.05 was considered statistically significant. Time-to-event analysis was limited to patients with complete paired admission dates. No Kaplan–Meier estimation was performed due to incomplete censoring information. No formal imputation was performed. Analyses were conducted using available-case methodology, and denominators were reported according to the number of complete observations for each variable.

Given the retrospective single-center design, the study is subject to residual confounding, referral bias, and incomplete capture of admissions occurring outside the institutional network. In addition, some second hospitalizations represented planned repeat interventions rather than unplanned readmissions. However, inclusion of consecutive patients, predefined eligibility criteria, and standardized data extraction were used to improve internal validity while preserving real-world clinical relevance. Complete paired admission and discharge dates were available only for a limited subgroup because many follow-up episodes originated from external regional referrals, incomplete archived records, or admissions documented without standardized longitudinal timestamp linkage in the retrospective database. Repeated observations within the same individuals were analyzed descriptively and were not modeled as fully independent longitudinal observations. Therefore, inferential comparisons across sequential episodes should be interpreted cautiously. Accordingly, *p*-values across sequential episodes should be interpreted cautiously and considered exploratory only.

## 3. Results

### 3.1. Baseline Characteristics

A total of 149 patients with cirrhosis were included in the study. The cohort was predominantly female (65.8%). The cohort predominantly underwent coil-based proximal or selective splenic artery embolization rather than particle-based parenchymal partial splenic embolization. Liver disease severity was most commonly Child–Pugh class B (49.0%), followed by class A (34.9%) and class C (16.1%), indicating a predominance of moderate hepatic dysfunction (Table 2).

The etiology of cirrhosis was predominantly viral. The largest subgroup consisted of HBV with HDV co-infection (27.5%), followed by HCV infection (26.2%) and HBV monoinfection (10.1%). Cholestatic liver diseases accounted for 12.1%, while autoimmune etiologies were present in 8.1% of patients.

Clinical manifestations of portal hypertension were highly prevalent. Esophageal varices were present in 85.1%, and ascites in 24.3% of patients at baseline. A history of variceal bleeding was documented in 7.4%.

Laboratory findings were consistent with hypersplenism and chronic liver disease, including thrombocytopenia (median 63 × 10^9^/L), leukopenia, and reduced albumin levels (median 35.0 g/L). Structural features of portal hypertension were common, including splenomegaly (88.5%), splenic vein dilatation (95.9%), and portal vein dilatation (95.2%).

### 3.2. Longitudinal Inpatient Episodes

**Figure 1:**
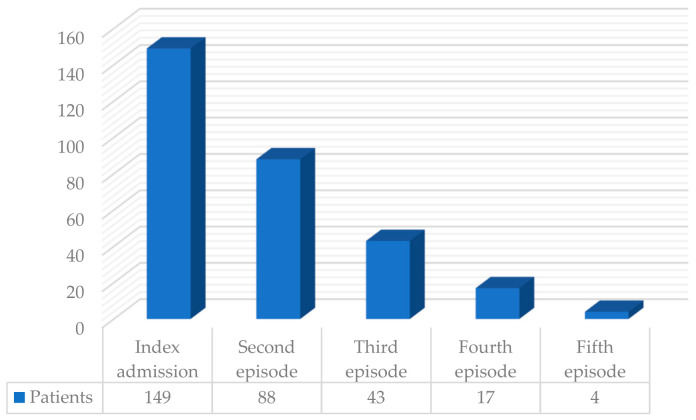
Sequential inpatient episodes following index splenic artery embolization.

Following index SAE, subsequent inpatient episodes were recorded, although progressively fewer patients contributed to later observations, reflecting variable observation time, referral-related loss of follow-up, possible mortality, transfer of care, and incomplete capture of hospitalizations outside the institutional database. (Figure 1).

Women comprised the majority of the cohort at baseline (65.8%) and remained predominant across subsequent recorded episodes. The sex distribution remained stable over time, suggesting no major sex-related differential retention (Table 3).

### 3.3. Liver Disease Severity During Follow-Up

At baseline, liver dysfunction was predominantly moderate. Child–Pugh class B was the most frequent category (49.0%), followed by class A (34.9%) and class C (16.1%) (Table 4). No statistically significant differences were observed across sequential episodes for Child–Pugh class A (*p* = 0.72), class B (*p* = 0.81), or class C (*p* = 0.65).

Overall, liver disease severity remained broadly stable among patients with repeated inpatient episodes.

### 3.4. Portal Hypertension Manifestations

Esophageal varices remained highly prevalent across all episodes, ranging from 75.0% to 87.8%, without significant variation (*p* = 0.48). Variceal bleeding was relatively infrequent, ranging from 0% to 11.6% (*p* = 0.41) (Table 5). Ascites was present in 24.3% at baseline, increased numerically at the second (35.0%) and third (35.9%) episodes, and decreased thereafter. This trend did not reach statistical significance (*p* = 0.09).

These findings indicate persistent portal hypertensive manifestations during follow-up, while overt bleeding events remained comparatively uncommon.

Markers of splenic congestion and portal venous remodeling remained highly prevalent. Splenomegaly was present in 84.6–100.0%, splenic vein dilatation in 83.3–100.0%, and portal vein dilatation in 84.6–100.0%. No significant longitudinal differences were observed (Table 6).

Structural features of portal hypertension remained largely unchanged across follow-up.

### 3.5. Hematologic and Biochemical Parameters

Platelet counts remained reduced throughout follow-up, with median values ranging from 55 to 68 × 10^9^/L, without significant longitudinal change (*p* = 0.58). White blood cell counts remained low (approximately 2.82–3.12 × 10^9^/L, *p* = 0.69), and red blood cell counts were stable (*p* = 0.64). Serum albumin demonstrated a numerical increase over time but did not reach statistical significance (*p* = 0.18) (Table 7).

Laboratory parameters demonstrated stability rather than progressive deterioration.

Exploratory analyses suggested that preserved liver function was more frequently observed among patients with relatively stable follow-up trajectories. Child–Pugh class A was more frequent among responders (45% vs. 30%, *p* = 0.04), whereas ascites was less frequent (20% vs. 40%, *p* = 0.03) (Table 8). Baseline albumin >35 g/L was also associated with a favorable outcome (60% vs. 35%, *p* = 0.02).

Patients with insufficient longitudinal follow-up information were not assigned to exploratory trajectory subgroup comparisons. Baseline variables were assessed during the index SAE hospitalization. Percentages were calculated within each subgroup.

### 3.6. Patterns and Timing of Subsequent Hospitalizations

**Figure 2:**
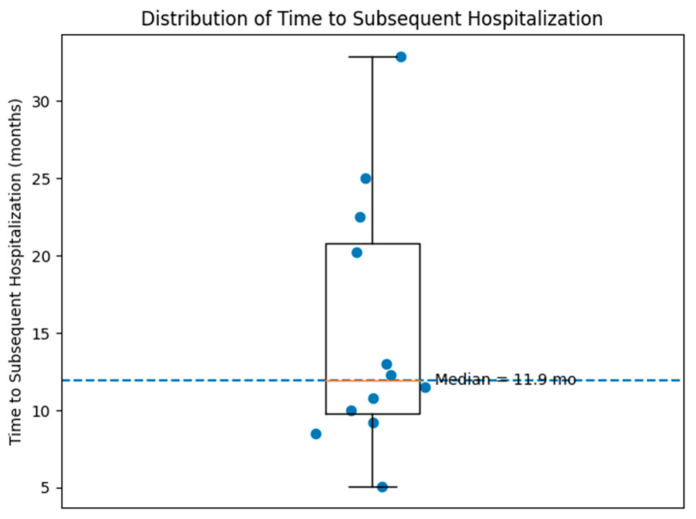
Distribution of time to subsequent hospitalization following splenic artery embolization among patients with available paired dates (*n* = 14). Individual circles represent patient-level observations. The box indicates the interquartile range, the horizontal line within the box represents the median value, whiskers indicate the minimum and maximum observed values, and the dashed horizontal line denotes the median time to subsequent hospitalization (11.9 months).

Among patients with available paired dates (*n* = 14), the median interval to the next hospitalization was 11.4 months (IQR 9.6–20.9). The mean interval was 14.8 ± 8.0 months, with a range of 5.1–32.9 months (Figure 2, Table 9). This analysis was restricted to patients with complete longitudinal data documentation and should therefore be interpreted cautiously because it reflects only a small subgroup of the overall cohort.

Individual data points are displayed to illustrate the distribution and variability of hospitalization intervals among patients with available follow-up data.

A total of 126 subsequent inpatient episodes were analyzed. The most common cause was ascites or hepatic decompensation (38.9%), followed by varices without bleeding (26.2%). Planned admissions for repeat SAE accounted for 7.9% of subsequent hospitalizations, reflecting the staged nature of treatment in a subset of patients. Other causes included hypersplenism (11.9%), variceal bleeding (7.9%), and procedure-related complications (7.1%) (Table 10).

No major procedure-related complications were documented in the available records. Minor post-embolization symptoms, including post-embolization syndrome, were not systematically recorded and therefore could not be reliably quantified in this retrospective cohort.

## 4. Discussion

### 4.1. Principal Interpretation

The present study provides real-world longitudinal data that may provide insights beyond short-term correction of cytopenia after splenic artery embolization in cirrhosis. In a cohort of 149 patients with portal hypertension and hypersplenism, one of the main descriptive observations was a progressive decrease in the number of recorded inpatient episodes over time, whereas long-term laboratory changes remained comparatively modest. This distinction is clinically relevant because, in advanced cirrhosis, recurrent inpatient care often reflects disease instability, decompensation risk, and healthcare burden more accurately than isolated hematologic indices [28,29].

Traditionally, SAE has been evaluated based on platelet response, leukocyte recovery, splenic volume reduction, or early technical success [30]. Our findings suggest that this framework may be too narrow. For many cirrhotic patients, fewer inpatient episodes, longer intervals between hospitalizations, and improved continuity of care may represent more meaningful outcomes than normalization of blood counts alone.

### 4.2. Hospitalization Burden as a Patient-Centered Outcome

Hospitalization is a major determinant of prognosis, healthcare cost, and quality of life in cirrhosis [31]. Recurrent inpatient episodes are commonly associated with infection risk, sarcopenia progression, renal dysfunction, treatment interruption, and accelerated functional decline [32]. Against this background, the observed decrease in the number of recorded inpatient episodes over time should be interpreted as a descriptive post-procedural trajectory rather than as evidence of a causal reduction in hospitalization burden attributable to SAE.

Importantly, subsequent inpatient episodes in this study included both planned and unplanned admissions, including repeat SAE procedures. Therefore, these findings should be interpreted as reflecting overall inpatient burden rather than strictly unplanned readmissions. Because causal inference cannot be established in a retrospective single-center study without a comparator group, multiple factors may have contributed to the observed pattern, including variable follow-up duration, regional referral logistics, admissions outside the institutional database, and underlying clinical heterogeneity.

Nevertheless, several clinically plausible mechanisms may explain why some patients experienced relatively prolonged intervals between recorded inpatient episodes following SAE. Reduction in splenic sequestration may facilitate outpatient management of cytopenia, reduce delays in procedures, decrease transfusion requirements, and improve tolerance of subsequent therapies [33]. In selected patients, partial modulation of portal hypertension may also contribute to greater clinical stability.

Among patients with complete paired longitudinal dates, the median interval to subsequent hospitalization was 11.4 months. Although this subgroup was limited in size, the finding suggests that some patients remained free from additional documented inpatient episodes for a clinically meaningful period following the index procedure. Future prospective studies should evaluate endpoints such as admission-free survival, days alive outside the hospital, and patient-reported outcomes.

### 4.3. Persistent Impact of the Underlying Liver Disease

Despite the decrease in the number of recorded inpatient episodes, the dominant causes of subsequent hospitalizations were ascites, hepatic decompensation, and variceal disease rather than procedure-related complications. This reinforces an important clinical principle: SAE addresses selected consequences of portal hypertension and hypersplenism but does not reverse the underlying liver disease process.

Progression of cirrhosis is driven by multiple interacting mechanisms, including worsening portal pressure, endothelial dysfunction, systemic inflammation, impaired synthetic function, and renal neurohormonal activation [34,35]. Therefore, even technically successful embolization should not be expected to prevent future decompensation in advanced disease.

Recent adjunctive strategies in cirrhosis have also focused on modulation of systemic inflammation and gut–liver interactions. For example, fecal microbiota transplantation has been associated with short-term improvement in hepatic encephalopathy severity, liver stiffness, steatosis, and inflammatory burden in selected patients with cirrhosis [36]. Although mechanistically distinct from SAE, these findings support the broader concept that supportive non-transplant interventions may improve patient-centered outcomes even without reversing the underlying cirrhotic process.

From a clinical perspective, SAE should therefore be considered an adjunctive supportive intervention rather than a disease-modifying therapy. Its role is likely to be most relevant when integrated into comprehensive longitudinal management, including etiologic treatment, endoscopic prophylaxis, nutritional support, and surveillance for complications [37].

### 4.4. Interpretation of the Hematologic Findings

One of the notable observations in this study was the absence of sustained statistically significant improvement in platelet or leukocyte counts at the cohort level. Although some previous studies have reported early hematologic improvement after splenic embolization, these effects appear variable across different embolization techniques and may attenuate over time [38,39].

Importantly, the present cohort predominantly underwent coil-based proximal or selective SAE rather than extensive particle-based parenchymal partial splenic embolization. This procedural distinction should be considered when comparing the present findings with prior PSE studies reporting larger infarction volumes and stronger hematologic responses.

Several explanations may account for the modest hematologic changes observed in this cohort. Thrombocytopenia in cirrhosis is multifactorial and may involve reduced thrombopoietin production, bone marrow suppression, chronic viral effects, alcohol-related toxicity, nutritional deficiencies, and inflammatory signaling pathways [39,40]. Similarly, leukopenia may reflect mechanisms beyond splenic sequestration alone. Accordingly, the absence of marked hematologic normalization should not necessarily be interpreted as the absence of clinical relevance. In patients with cirrhosis, clinically meaningful effects may still occur despite persistently abnormal laboratory parameters.

### 4.5. Importance of Timing and Patient Selection

Baseline hepatic reserve appeared to influence clinical trajectories. Patients with better preserved liver function and higher albumin levels were more frequently observed among those with a favorable course, whereas ascites and a more advanced Child–Pugh stage were more common in patients with less favorable outcomes.

This observation is consistent with broader cirrhosis literature, where interventions tend to be more effective before advanced decompensation develops [41,42]. Once complications such as refractory ascites, renal dysfunction, and frailty are established, correction of hypersplenism alone may have a limited impact on the overall trajectory. These findings support earlier and more selective use of SAE rather than restricting it to late-stage settings. In clinical practice, the key question may not be whether SAE improves platelet counts but whether it is applied at a stage where meaningful benefit is still achievable.

### 4.6. Structural Disease Versus Functional Outcomes

Markers of portal hypertension, including varices, splenomegaly, and venous dilatation, remained prevalent during follow-up. This suggests that clinical stabilization may occur without reversal of structural abnormalities.

A similar dissociation between anatomical persistence and clinical benefit has been described in other portal hypertension interventions [36,42,43]. Therefore, lack of radiologic normalization should not be equated with lack of therapeutic effect.

### 4.7. Clinical Implications

For multidisciplinary teams, realistic objectives of SAE may include:

- reduction in overall inpatient burden;
- mitigation of hypersplenism-related treatment limitations;
- improved tolerance of planned procedures or therapies;
- selective stabilization in appropriately chosen patients.

In exploratory analyses, patients with preserved or moderately impaired liver function more frequently demonstrated relatively stable follow-up trajectories. In contrast, expectations should remain cautious in patients with advanced multisystem decompensation [44].

### 4.8. Value of the Present Study

The value of this study lies in its real-world longitudinal characterization of patients with cirrhosis, portal hypertension, and hypersplenism treated with SAE in a tertiary referral center in Kazakhstan. While many studies of splenic embolization focus primarily on short-term technical success or early laboratory response, the present cohort reflects routine clinical practice, variable regional follow-up, repeated inpatient episodes, and the challenges of monitoring patients referred from geographically distant areas.

Although the absence of an untreated or matched control group precludes causal conclusions, the study provides clinically relevant information on longitudinal inpatient trajectories, hematologic trends, documented variceal bleeding, repeat SAE procedures, and major procedure-related safety outcomes.

These findings may help inform future prospective studies and support the development of more standardized outcome measures, including platelet and leukocyte response dynamics, systematic post-embolization syndrome reporting, infarction-volume assessment, clinically driven readmissions, and patient-centered longitudinal endpoints.

### 4.9. Limitations

Several limitations should be acknowledged. The retrospective single-center design limits causal inference and introduces potential residual confounding. The decreasing number of patients contributing to later follow-up episodes likely reflects a combination of heterogeneous observation time, possible mortality, transfer of care, and incomplete capture of hospitalizations occurring outside the study center.

Importantly, subsequent hospitalizations included both planned and unplanned admissions and therefore should not be interpreted as strictly unplanned readmission events. This should be considered when interpreting longitudinal hospitalization patterns. Laboratory data were not uniformly available at all time points, and repeated measurements within individuals were not formally adjusted for in the statistical analysis. In addition, no matched control group was available for comparison. Baseline demographic variables, such as age, were incompletely available and therefore were not included in the analysis. These limitations restrict causal interpretation but do not diminish the descriptive value of the observed real-world clinical trajectories.

Post-embolization syndrome was considered an expected minor post-procedural reaction after SAE rather than a major complication. However, because these symptoms were not systematically recorded using standardized criteria in retrospective medical records, the true frequency of minor post-procedural complications could not be reliably assessed in this cohort. Therefore, safety conclusions were limited to documented major complications and clinically significant procedure-related events available in the medical records.

The safety analysis focused on documented major complications and clinically significant procedure-related events. No splenic abscess, portal or splenic vein thrombosis, pancreatitis, pleural effusion, clinically significant infection, procedure-related mortality, or need for urgent surgical intervention was documented in the available records.

## 5. Conclusions

This retrospective single-center before–after study provides real-world longitudinal data on patients with cirrhosis, portal hypertension, and hypersplenism undergoing predominantly coil-based proximal or selective splenic artery embolization in a tertiary referral setting. The study demonstrates that SAE was feasible in a clinically complex cohort and was not associated with documented major procedure-related complications such as splenic abscess, portal or splenic vein thrombosis, pancreatitis, urgent surgical intervention, or procedure-related mortality in the available records.

The main measurable post-procedural findings were reflected in hematologic trajectories, primarily platelet and leukocyte dynamics, rather than in validated composite clinical endpoints. Documented variceal bleeding after SAE was relatively uncommon during follow-up, whereas later inpatient episodes mainly reflected the ongoing burden of cirrhosis and portal hypertension rather than procedure-related complications.

The value of this study lies in its real-world characterization of post-SAE follow-up in an underrepresented regional setting, where patients are frequently referred from geographically distant regions and long-term standardized follow-up remains challenging. These findings may support consideration of SAE as a minimally invasive adjunctive option in selected patients with cirrhosis-associated hypersplenism while emphasizing the need for careful patient selection, standardized reporting of post-embolization syndrome, systematic assessment of infarction volume, and prospective controlled studies to better define long-term clinical outcomes.

## Figures and Tables

**Table 1:** The inclusion and exclusion criteria.

Domain	Inclusion Criteria	Exclusion Criteria
Age	≥18 years	<18 years
Diagnosis	Confirmed liver cirrhosis of any etiology	Absence of verified cirrhosis
Intervention	Underwent SAE during study period	Embolization for trauma, aneurysm, malignancy, or other non-cirrhotic indications
Documentation	Complete index hospitalization and procedural records	Missing treatment date or absent core procedural documentation
Data quality	Verifiable baseline clinical characteristics	Duplicate, conflicting, or irreconcilable records
Follow-up	At least one traceable outcome entry or institutional follow-up record	Insufficient data for outcome assessment

**Table 2:** Baseline characteristics of the study cohort (*n* = 149).

Variable	Value
Sex, *n* (%)	
Female	98 (65.8)
Male	51 (34.2)
Etiology of cirrhosis, *n* (%)	
HBV + HDV	41 (27.5)
HCV	39 (26.2)
HBV	15 (10.1)
Cholestatic (PBC/PSC)	18 (12.1)
Autoimmune (AIH ± overlap)	12 (8.1)
Toxic	11 (7.4)
Metabolic (MAFLD)	5 (3.4)
Cryptogenic/other	8 (5.4)
Child–Pugh class, *n* (%)	
Class A	52 (34.9)
Class B	73 (49.0)
Class C	24 (16.1)
Ascites, *n* (%)	36 (24.3)
Varices, *n* (%)	127 (85.1)
Prior bleeding, *n* (%)	11 (7.4)
Platelets, median (IQR), ×10^9^/L	63 (52–84)
WBC, median (IQR), ×10^9^/L	3.01 (2.33–3.90)
RBC, median (IQR), ×10^12^/L	4.07 (3.66–4.38)
Albumin, median (IQR), g/L	35.0 (31.2–38.7)
Splenomegaly, *n* (%)	132 (88.5)
Splenic vein dilatation, *n* (%)	142 (95.9)
Portal vein dilatation, *n* (%)	142 (95.2)

**Table 3:** Sex distribution across sequential episodes.

Episode	Total *n*	Female *n* (%)	Male *n* (%)
Index admission	149	98 (65.8)	51 (34.2)
Second episode	88	56 (63.6)	32 (36.4)
Third episode	43	28 (65.1)	15 (34.9)
Fourth episode	17	11 (64.7)	6 (35.3)
Fifth episode	4	3 (75.0)	1 (25.0)

**Table 4:** Child–Pugh class across sequential episodes.

Variable	Baseline (%)	Second (%)	Third (%)	Fourth (%)	Fifth (%)	*p*-Value
Child A	34.9	31.0	34.8	29.4	50.0	0.72
Child B	49.0	53.6	45.7	52.9	50.0	0.81
Child C	16.1	15.5	19.6	17.7	0.0	0.65

**Table 5:** Portal hypertension features across sequential episodes.

Variable	Baseline (%)	Second (%)	Third (%)	Fourth (%)	Fifth (%)	*p*-Value
Esophageal varices	85.1	78.3	87.8	81.3	75.0	0.48
Variceal bleeding	7.4	8.0	11.6	5.9	0.0	0.41
Ascites	24.3	35.0	35.9	26.7	25.0	0.09

**Table 6:** Structural imaging findings.

Variable	Baseline (%)	Second (%)	Third (%)	Fourth (%)	Fifth (%)	*p*-Value
Splenomegaly	88.5	87.1	89.2	84.6	100.0	0.77
Splenic vein dilatation	95.9	93.9	88.6	83.3	100.0	0.32
Portal vein dilatation	95.2	94.1	91.7	84.6	100.0	0.28

**Table 7:** Laboratory parameters across sequential episodes.

Variable	Baseline	Second	Third	Fourth	Fifth	*p*-Value
Platelets, median (IQR), ×10^9^/L	63 (52–84)	60 (47–82.8)	64 (41.5–80.5)	68 (55–80)	55 (37–73)	0.58
WBC, median (IQR), ×10^9^/L	3.01 (2.33–3.90)	2.90 (2.31–3.70)	3.12 (2.09–3.94)	3.13 (2.32–3.86)	2.82 (2.14–3.64)	0.69
RBC, median (IQR), ×10^12^/L	4.07 (3.66–4.38)	4.01 (3.68–4.39)	3.99 (3.66–4.41)	4.29 (3.88–4.64)	3.84 (3.71–4.06)	0.64
Albumin, median (IQR), g/L	35.0 (31.2–38.7)	35.8 (28.0–38.7)	36.6 (32.3–38.5)	35.0 (30.4–38.2)	38.0 (31.2–42.5)	0.18

**Table 8:** Exploratory baseline characteristics according to documented follow-up trajectory.

Variable	Relatively Stable Documented Follow-Up Trajectory (*n* = 41)	Less Stable Documented Follow-Up Trajectory (*n* = 27)	*p*-Value
Child–Pugh A, *n* (%)	19 (46.3)	8 (29.6)	0.04
Child–Pugh B/C, *n* (%)	22 (53.7)	19 (70.4)	0.04
Ascites present, *n* (%)	8 (19.5)	11 (40.7)	0.03
Albumin > 35 g/L, *n* (%)	25 (61.0)	10 (37.0)	0.02
Esophageal or gastric varices, *n* (%)	29 (70.7)	23 (85.2)	0.08
Severe splenomegaly on imaging, *n* (%)	33 (80.5)	24 (88.9)	0.21

**Table 9:** Time to Subsequent Hospitalization.

Parameter	Value
*N*	14
Mean ± SD	14.8 ± 8.0 months
Median (IQR)	11.4 (9.6–20.9)
Range	5.1–32.9

**Table 10:** Causes of subsequent hospitalization.

Cause	*n* (%)
Ascites/hepatic decompensation	49 (38.9)
Varices without bleeding	33 (26.2)
Hypersplenism	15 (11.9)
Variceal bleeding	10 (7.9)
Repeat SAE (planned admission)	10 (7.9)

## Data Availability

The datasets analyzed during the current study are not publicly available due to institutional and patient privacy restrictions but are available from the corresponding author on reasonable request.

## Abbreviations

The following abbreviations are used in this manuscript:

SAE	Splenic artery embolization
HBV	Hepatitis B virus
HCV	Hepatitis C virus
HDV	Hepatitis D virus
AIH	Autoimmune hepatitis
PBC	Primary biliary cholangitis
PSC	Primary sclerosing cholangitis
MAFLD	Metabolic dysfunction-associated fatty liver disease
WBC	White blood cell count
RBC	Red blood cell count
PLT	Platelet count
MELD	Model for End-Stage Liver Disease
